# The Rate of Clinical Outcomes in Atrial Fibrillation according to Antithrombotic Strategy: The COOL-AF Registry

**DOI:** 10.1155/2022/5797257

**Published:** 2022-02-21

**Authors:** Rungroj Krittayaphong, Arjbordin Winijkul, Komsing Methavigul, Poom Sairat, C. O. O. L.-A. F. investigators

**Affiliations:** ^1^Division of Cardiology, Department of Medicine, Faculty of Medicine Siriraj Hospital, Mahidol University, Bangkok, Thailand; ^2^Department of Cardiology, Central Chest Institute of Thailand, Nonthaburi, Thailand; ^3^Her Majesty Cardiac Center, Mahidol University, Bangkok, Thailand

## Abstract

**Background:**

Ischemic stroke/transient ischemic attack (TIA), major bleeding, and death are common outcomes in atrial fibrillation (AF) patients, so appropriate antithrombotic therapy is crucial. The objective of this study was to investigate the rate of ischemic stroke/TIA, major bleeding, and death compared among AF patients who received oral anticoagulant (OAC) alone, antiplatelet alone, or OAC plus antiplatelet.

**Methods:**

Prospective data from the COOL-AF Registry (Thailand's largest multicenter nationwide AF registry) were analyzed. Clinical, laboratory, and medication data were collected at baseline and during follow-up. Clinical outcomes, including ischemic stroke/TIA, major bleeding, and death, were collected.

**Results:**

There were 3,148 patients included. Mean age was 68.1 ± 10.8 years and 1,826 (57.7%) were male. AF was paroxysmal in 998 (31.7%), persistent in 603 (19.2%), and permanent in 1,547 (49.1%). The mean follow-up duration was 25.7 ± 10.6 months. The median rates of ischemic stroke/TIA, major bleeding, and death were 1.49 (1.21-1.81), 2.29 (1.94-2.68), and 3.89 (3.43-4.40) per 100 person-years. Antiplatelet alone, OAC plus antiplatelet, and OAC alone were used in 582 (18.5%), 308 (9.8%), and 2,258 (71.7%) patients, respectively. Antiplatelet alone significantly increased the risk of ischemic stroke/TIA and death compared to OAC alone. OAC plus antiplatelet significantly increased the risk of death compared to OAC alone.

**Conclusions:**

Antiplatelet was used in 890 (28.3%) AF, of whom 582 (18.5%) received antiplatelet alone, and 308 (9.8%) received antiplatelet and OAC. OAC plus antiplatelet significantly increased the risk of death without additional stroke prevention benefit. Antiplatelet alone should not be used in patients with AF.

## 1. Introduction

Ischemic stroke is a major complication in patients with nonvalvular atrial fibrillation (AF) [1]. Oral anticoagulant (OAC) is recommended in patients who are not in the low-risk category [2]. In the CHA_2_DS_2_-VASc scoring system, vascular disease is defined as significant coronary artery disease (CAD), previous myocardial infarction (MI), peripheral arterial disease (PAD), or aortic plaque [3]. Antiplatelet alone is not recommended for stroke prevention in patients with AF [2, 4, 5]. There exists a common misconception that antiplatelet can be used for stroke prevention in AF [6].

Antiplatelet helps to prevent cardiovascular events in patients with CAD [7]; however, OAC without antiplatelet is recommended in AF patients who have stable CAD [2, 7]. For those with AF and recent acute coronary syndrome (ACS) or percutaneous coronary intervention (PCI), OAC is recommended in combination with a short duration of dual antiplatelet followed by OAC and single antiplatelet for up to 12 months [8]. Previous studies showed that OAC plus antiplatelet significantly increased the risk of major bleeding [9]. OAC plus dual antiplatelet increased the risk of major bleeding more than OAC plus single antiplatelet without conferring added ischemic stroke reduction benefit [10]. Therefore, physicians must compare the risks and benefits between antiplatelet alone and antiplatelet in combination with OAC in patients with AF [9].

The aim of this study was to investigate the rate of ischemic stroke/TIA, major bleeding, and death in patients in the COhort of antithrombotic use and Optimal INR Level in patients with nonvalvular Atrial Fibrillation in Thailand (COOL-AF) Registry compared among those who received oral OAC alone, antiplatelet alone, or OAC plus antiplatelet.

## 2. Methods

### 2.1. Study Population

We enrolled consecutive patients with electrocardiograph- (ECG-) confirmed nonvalvular AF (NVAF) with age at least 18 years from 27 hospitals in Thailand. The protocol for this study was approved by the Institutional Review Board (IRB) of each participating hospital. All patients gave written informed consent prior to participation. Patients with at least one of the following were excluded: (1) rheumatic mitral stenosis; (2) prosthetic heart valve; (3) AF from transient reversible cause; (4) current participation in a clinical trial; (5) life expectancy less than 3 years; (6) bleeding disorders, such as thrombocytopenia or myeloproliferative disorders; (7) pregnancy; (8) refusal to participate; (9) ischemic stroke within 3 months; or (10) inability to attend follow-up. Those who did not receive either OAC or antiplatelet were also excluded since these are low-risk patients that should not be compared with AF patients that are not considered low risk.

### 2.2. Study Protocol

Details specific to this cohort were previously described [11]. Investigators obtained patient data from the medical record and patient interview. Investigators recorded the required data in a study-specific case record form (CRF), and a web-based system was used to collect CRF data.

Patients were followed up at 6, 12, 18, 24, and 30 months after enrollment. Data recording and verification was performed in the same manner as it was performed at the baseline visit.

Site monitoring was performed at every study site to audit the data collection and recording processes and to ensure compliance with the Good Clinical Practice (GCP) guideline.

### 2.3. Data Collection

Collected data included demographic variables, vital signs, symptom and type of AF, complication of AF, past medical history, previous treatment for AF, current medications for AF, and other medications. Each component of the CHA_2_DS_2_-VASc score was scored and recorded, as follows: C = congestive heart failure (1 point), H = hypertension (1 point), A = age > 75 years (2 points), D = diabetes (1 point), S = stroke (2 points), V = vascular disease (1 point), A = age 65-74 (1 point), and Sc = female sex category (1 point). Each component of the HAS-BLED score was scored and recorded, as follows: uncontrolled hypertension, abnormal renal, or liver function; history of stroke; history of bleeding; labile INR; elderly (age above 65 years); and drugs or alcohol (1 point each). During each follow-up visit, special care was taken to determine if any of the 3 study outcomes occurred during the preceding six-month period.

### 2.4. Outcomes

The clinical outcomes were ischemic stroke/TIA, major bleeding, and death. The definition of ischemic stroke was sudden-onset neurologic deficit lasting longer than 24 hours. The duration of the neurologic deficit was less than 24 hours for TIA. Major bleeding was defined according to International Society of Thrombosis and Hemostasis (ISTH) criteria [12]. The source document for verification of clinical outcomes was uploaded into the web-based system. All source documents, including imaging for those who had stroke or TIA, were sent to the adjudication committee for confirmation of the clinical outcome.

### 2.5. Statistical Analysis

Continuous data are described as mean plus/minus standard deviation (SD). Comparisons of continuous data between 2 groups were made using Student's *t*-test for unpaired data. Continuous data among 3 groups were compared using analysis of variance (ANOVA). Post hoc analysis of the ANOVA test was performed using Bonferroni method. Categorical data are described as number and percentage. Comparisons of categorical data were made using chi-square test or Fisher's exact test. Univariate and multivariate Cox proportional hazard models were used to determine the time-dependent effect of antithrombotic regimens on the clinical outcomes. The following variables were used as covariates in the multivariate model: age, gender, AF type and symptom, hypercholesterolemia, hypertension, current smoker status, history of ischemic stroke, CAD, heart failure, history of major bleeding, cardiac implantable electronic device (CIED), and renal replacement therapy. Kaplan-Meier plot was used to display the cumulative event rate during follow-up. The antithrombotic regimens were classified as (1) OAC alone, (2) antiplatelet alone, and (3) OAC plus antiplatelet. The Cox model was also used to calculate the rate of clinical outcome with “death without event” as a competing risk. Since the proportion of patients receiving dual antiplatelet in this cohort was small, we did not endeavor to compare differences in clinical outcome between those taking double platelet and those taking single platelet. Sensitivity analysis was performed to determine the effect of antithrombotic regimens on clinical outcomes in high-risk subset (men with CHA_2_DS_2_-VASc score ≥ 2 and women with CHA_2_DS_2_-VASc score ≥ 3). All analyses were performed using SPSS Statistics software version 18.0 (SPSS, Inc., Chicago, IL, USA). A *p* value of less than 0.05 was considered statistically significant.

## 3. Results

### 3.1. Baseline Characteristics

**Figure 1:**
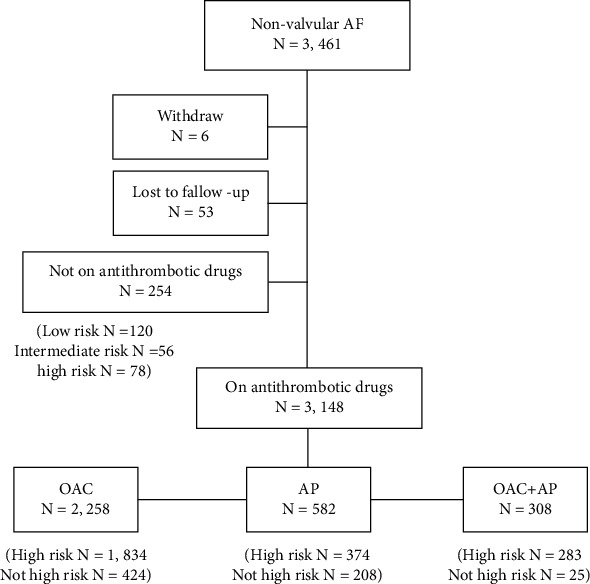
Flow diagram of study population (OAC: oral anticoagulant; AP: antiplatelet).

The entire cohort consisted of 3,461 patients. However, follow-up data was not available in 59 patients, and 254 patients did not receive antithrombotic medication, so data from 3,148 patients were included in our analysis. The flow diagram of the study population is shown in Figure 1. The average age of patients was 68.1 ± 10.8 years, and 1,826 (57.7%) were male. Antithrombotic regimen was OAC alone in 2,258 (71.7%), antiplatelet alone in 582 (18.5%), and OAC plus antiplatelet in 308 (9.8%) patients. Among 2,566 patients who were on OAC, 2,338 (91.1%) and 228 (8.9%) patients received warfarin and non-vitamin K antagonist oral anticoagulant (NOAC), respectively. Among 890 patients who were on antiplatelet, 784 (88.1%) were on aspirin and 200 (22.5%) were taking P2Y12 inhibitors. Clopidogrel was prescribed in 194 patients (97% of P2Y12 inhibitors). Dual antiplatelet was used in 97 patients (10.9%). Dual antiplatelet was used in combination with OAC in 38 patients (4.3%). Baseline clinical characteristics of the 3 groups are shown in Table 1.

### 3.2. Relation of Antiplatelet Use and History of CAD and PAD

Among 3,148 patients, history of CAD, ischemic stroke/TIA, and PAD were present in 540 (17.2%), 578 (18.4%), and 41 (1.3%) patients, respectively. A total of 566 (18.0%) patients had history of CAD or PAD. Among 890 patients who received antiplatelet agent, only 73 (8.2%) had history of ACS or PCI within 1 year, which was considered appropriate use of antiplatelet. Of those 890 patients, 313 (35.2%), 101 (11.3%), and 21 (2.4%) patients had history of CAD, ischemic stroke/TIA, and PAD, respectively. Among patients with stable CAD, 227 (42.0%), 124 (23.0%), and 189 (35.0%) received OAC alone, antiplatelet alone, and OAC plus antiplatelet, respectively. Among patients with ACS or PCI within 1 year, 27 (27.0%), 33 (33.0%), and 40 (40.0%) received OAC alone, antiplatelet alone, and OAC plus antiplatelet, respectively.

### 3.3. Association of Antithrombotic Regimen with Clinical Outcomes

**Figure 2:**
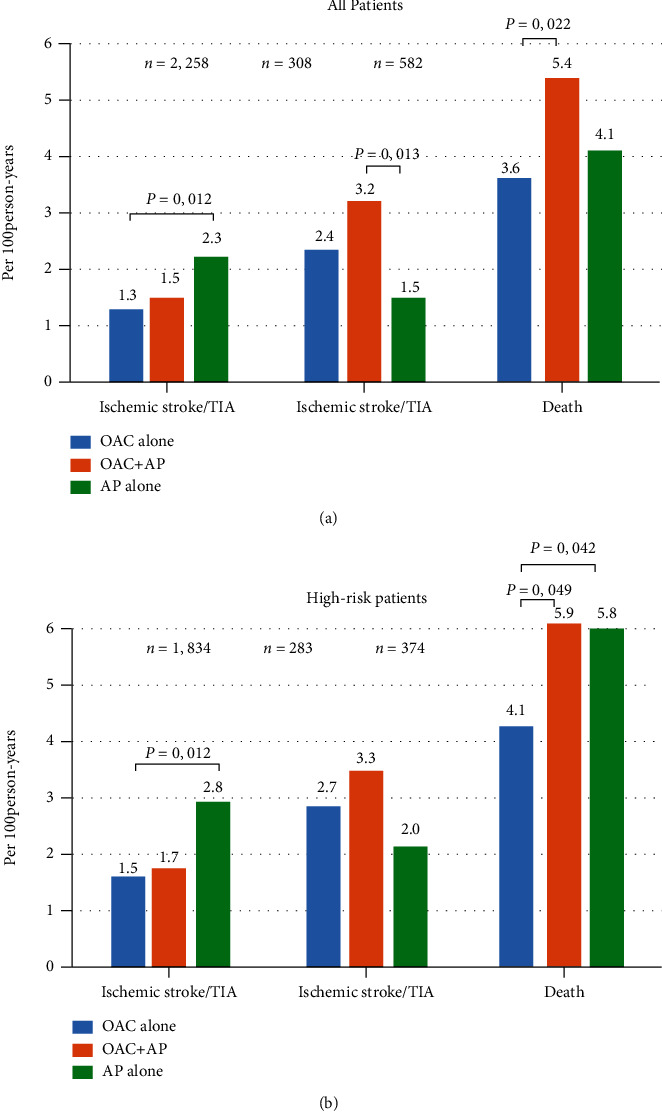
Incidence rate of ischemic stroke/transient ischemic attack (TIA), major bleeding, intracerebral hemorrhage (ICH), and death compared among patients with oral anticoagulant (OAC) alone, OAC plus antiplatelet, and antiplatelet alone: (a) all patients; (b) high-risk patients.

The average follow-up duration was 25.7 ± 10.6 months (6,651.5 person-years). The median rate of ischemic stroke/TIA, major bleeding, and death was 1.49 (1.21-1.81), 2.29 (1.94-2.68), and 3.89 (3.43-4.40) per 100 person-years, respectively. The rate of clinical outcomes in each antithrombotic regimen group is shown in Table 2.  Figure 2 compares the incidence rate of clinical outcomes among patients who received OAC alone, antiplatelet alone, and OAC plus antiplatelet both for the whole group and in the high-risk group. The high-risk group was defined as male with CHA_2_DS_2_‐VASc ≥ 2 or female with CHA_2_DS_2_‐VASc ≥ 3. The rate of ischemic stroke/TIA was highest in patients who received antiplatelet alone. The rate of major bleeding and death was highest in those who received OAC plus antiplatelet. In the high-risk group, the death rate of antiplatelet alone and OAC plus antiplatelet was significantly higher than that of OAC alone.

Among 2,338 patients who were on warfarin, 2,295 (98.2%) had enough international normalized ratio (INR) data to calculate time in therapeutic range (TTR). TTR was calculated using the Rosendaal method [13]. The average TTR was 53.6 ± 26.4%. The TTR of patients on OAC alone was significantly higher than that of patients taking OAC plus antiplatelet 54.2 ± 26.2% vs. 48.8 ± 27.0% (*p* = 0.001).

### 3.4. Kaplan-Meier Analysis

**Figure 3:**
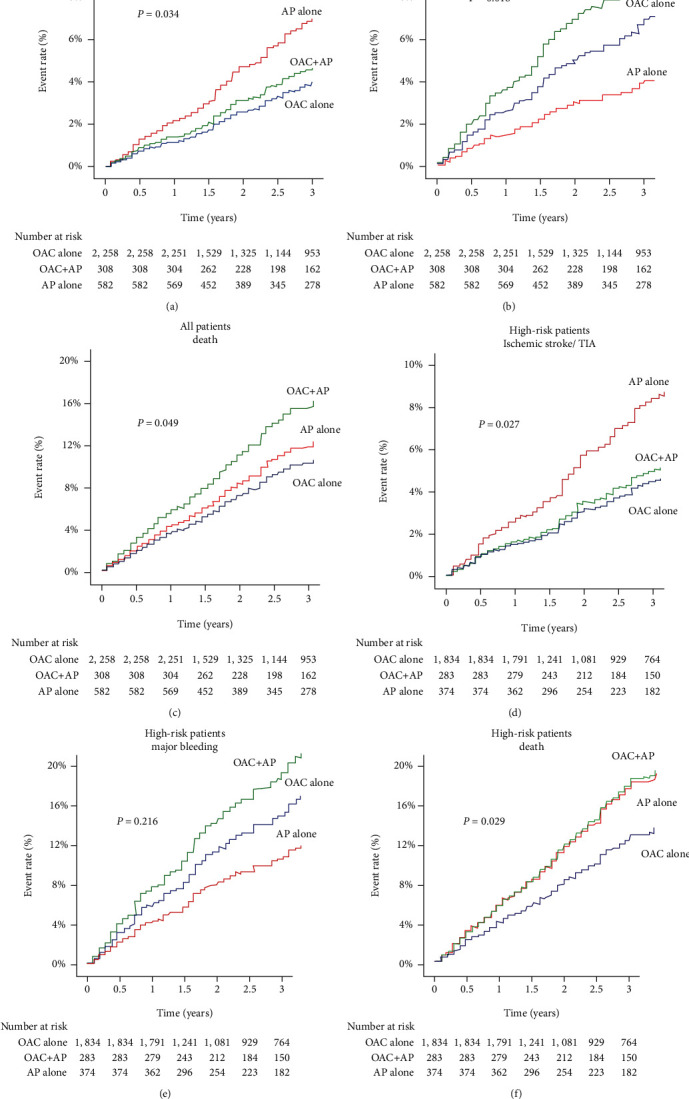
(a) Cumulative event rate over time for ischemic stroke/transient ischemic attack (TIA), (b) major bleeding, (c) death among patients with oral anticoagulant (OAC) alone, OAC plus antiplatelet, and antiplatelet alone.

Figure 3 demonstrates the cumulative event rate over time for ischemic stroke/TIA, major bleeding, and death compared among patients receiving OAC alone, OAC plus antiplatelet, or antiplatelet alone for the whole group and in the high-risk group. Both crude and adjusted Cox-proportional hazard model showed significant difference in ischemic stroke/TIA, major bleeding, and death among antithrombotic regimen groups. Ischemic stroke/TIA in patients receiving antiplatelet alone was higher than that in the other 2 groups. Major bleeding was highest in patients on OAC plus antiplatelet, followed by OAC alone, and antiplatelet alone. Death was highest in patients taking OAC plus antiplatelet. The results shown in Figure 3 support the finding in Figure 2.

### 3.5. Univariate and Multivariate Analysis

**Figure 4:**
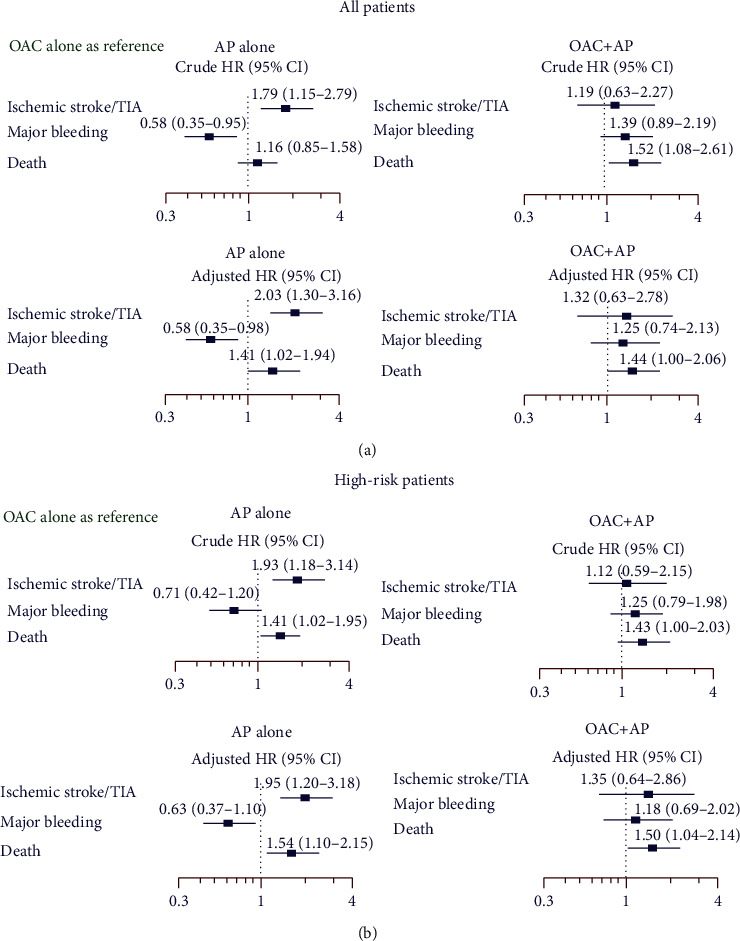
Forest plot demonstrates forest plot of crude and adjusted hazard ratio (HR) and 95% confidence interval (CI) of effect of antiplatelet (AP) alone and oral anticoagulant (OAC) plus AP compared to OAC alone on ischemic stroke/transient ischemic attack (TIA), major bleeding, and death: (a) all patients; (b) high-risk patients.

Figure 4 shows a forest plot of the crude hazard ratio (HR), 95% confidence interval (CI), and adjusted HR (95% CI) compared among ischemic stroke/TIA, major bleeding, and death for antiplatelet alone versus OAC alone (reference) and for OAC plus antiplatelet versus OAC alone (reference) in all patients and in high-risk patients. The following variables were used as covariates in the multivariate model: age, gender, AF type and symptom, hypercholesterolemia, hypertension, current smoker status, history of ischemic stroke, CAD, heart failure, history of major bleeding, CIED, and renal replacement therapy.

Multivariate analysis also showed that antiplatelet alone increased the risk of ischemic stroke/TIA and death compared to OAC alone. OAC plus antiplatelet increased the risk of death compared to OAC alone both in univariate and in multivariate analyses. OAC plus antiplatelet had no additional benefit over OAC alone for the prevention of ischemic stroke/TIA.

### 3.6. Sensitivity Analysis

Univariate and multivariate analyses of patients in the high-risk category were performed to evaluate the effect of antithrombotic regimen on clinical outcomes. Antiplatelet alone increased the risk of ischemic stroke/TIA and death compared to OAC alone in both univariate and multivariate analyses. OAC plus antiplatelet increased the risk of death compared to OAC alone in both univariate and multivariate analyses. The analysis was also performed with adjustment for time-varying covariates, and the results of those analyses were not different from those from the main analyses.

## 4. Discussion

The results of this prospective multicenter nationwide AF registry revealed the use of antiplatelet in 890 (28.3%) patients with AF. Of those, antiplatelet alone was used in 582 (18.5%) patients and antiplatelet plus OAC was prescribed in 308 (9.8%) patients. Patients who received antiplatelet alone or OAC plus antiplatelet had an increased risk of adverse outcome compared to those receiving OAC alone.

The rate of using antiplatelet alone in our study was 18.5%, which is comparable to data from the Global Anticoagulant Registry in the FIELD (GARFIELD) Registry [14], which demonstrated a temporal change in the practice of antithrombotic use. Our rate was also similar to the results of the EURObservational Research Programme Atrial Fibrillation (EORP-AF) study. From the GARFIELD study, the rate of using antiplatelet alone decreased in the later cohort compared to the earlier cohort [14]. The rate of using antiplatelet alone in the Asian population was higher than in our study. Approximately 30% of patients with AF in the FUSHIMI study used antiplatelet alone [15]. The rate of antiplatelet alone was even higher in a Chinese population with AF, which was more than 50% [16]. The high rate of using antiplatelet alone and the low rate of using OAC in the Asian population might be related to a fear of bleeding complication [17]. In addition to the rate of OAC use being lower in the Asian population, the TTR was also lower in the Asian population compared to the Caucasian population [18]. Bleeding complication was reported to be higher in the Asian population when they were on anticoagulant, especially warfarin [19, 20]. Among 582 patients who received antiplatelet alone in our study, 131 (22.5%) had CAD or PAD. It is possible that physicians were reluctant to change antiplatelet to OAC in patients with CAD and AF [21]. We demonstrated that, although the major bleeding rate in the antiplatelet alone group was lower than that in the OAC alone group, the stroke rate in the antiplatelet alone group was significantly higher than that in the OAC alone group. We also showed that the antiplatelet alone group had a significantly higher rate of death compared to the OAC alone group.

Previous studies reported that combination OAC and antiplatelet may harm patients due to an increased risk of major bleeding, including intracerebral hemorrhage (ICH) [9, 22]. Therefore, OAC plus antiplatelet should be used in patients with appropriate indications, such as AF patients with recent ACS or PCI less than one year [8]. In our study 9.8% of AF patients received OAC plus antiplatelet. Among 308 patients with OAC plus antiplatelet, only 40 (13.0%) patients had recent ACS or PCI. Moreover, 192 (62.3%) of them had a history CAD or PAD. Therefore, a misperception may exist among physicians that when CAD or PAD patients have AF, they should continue using antiplatelet despite the use of OAC [9, 21]. The rate of major bleeding was significantly higher in patients taking OAC plus antiplatelet compared to those who receiving OAC alone [22]. Results from the PREvention oF thromboembolic events–European Registry in Atrial Fibrillation (PREFER in AF) that enrolled 1,058 patients with AF and stable CAD (more than 1 year after ACS or PCI) demonstrated that OAC plus antiplatelet had no stroke prevention benefit compared to OAC alone, and that OAC plus antiplatelet was associated with increased risk of major bleeding [23]. In our study, the major bleeding rate increased from 4.9% in the OAC alone group to 7.5% in OAC plus antiplatelet group over a follow-up duration of 25.7 months, but the observed increase was not statistically significant. In our study, the rate of death was significantly higher in the OAC plus antiplatelet group than in the OAC alone group. The major bleeding rate in our study could be higher if physicians consider INR control in patients with OAC plus antiplatelet similar to the OAC alone group. The TTR in the OAC plus antiplatelet group in our study was significantly lower than that in the OAC alone group. This may be due to the fact that physicians tend to maintain a lower INR in patients on OAC plus antiplatelet when compared to patients taking OAC alone, which might be related to a fear of bleeding when they prescribed both drugs together [21]. This finding is similar to that from a previous report on the effect of aspirin on warfarin control in 4,494 patients taking warfarin [24]. They found a lower TTR when warfarin was combined with aspirin compared to warfarin alone. Poor TTR control has been shown to be an indicator for the prediction of adverse clinical outcome [25]. In our study, adding antiplatelet to OAC did not provide better protection against ischemic stroke/TIA compared to OAC alone. This may be partially explained by the lower TTR in the combination group compared to the OAC alone group. Major guidelines recommend that in AF patients with stable CAD, OAC should be used alone to avoid the unnecessary risk of major bleeding [2, 4, 5]. However, the rate of inappropriate combination of antithrombotics remains high [15, 21].

The clinical implication of the results of this study is that improved antithrombotic therapy guideline adherence is needed among physicians treating patients with AF. Previous studies showed that patients who were on guideline-recommended antithrombotic regimen had better outcomes that those whose treatment was not consistent with guideline recommendations [26]. Following guideline recommendations, the antiplatelet should not be used alone and should not be used in combination with OAC unless patients have an appropriate indication for combination therapy. Previous studies demonstrated that AF patients who were treated for stroke prevention according to guideline recommendation had better clinical outcomes than those who did not follow guideline recommendation [2, 27]. Awareness of and knowledge about anticoagulant use in patients with AF and CAD should be improved.

The notable limitation of this study is that our registry cohort included only a small proportion of patients who received OAC plus antiplatelet (9.8%). This may have limited the power of our study to identify all significant associations and differences specific to this group when compared to other groups in our study. Another limitation is that we did not collect the reasons for using antiplatelet drugs. Therefore, despite the fact that only small proportion of patients had a history of ACS or receiving PCI within 1 year and antiplatelet use increased risk of adverse outcomes compared to OAC, we cannot definitely conclude that majority of patients had an inappropriate use of antiplatelet drugs.

## 5. Conclusion

Antiplatelet was used in 890 (28.3%) AF, of whom 582 (18.5%) received antiplatelet alone, and 308 (9.8%) received antiplatelet and OAC. OAC plus antiplatelet significantly increased the risk of death without additional stroke prevention benefit. Antiplatelet alone should not be used in patients with AF.

## Figures and Tables

**Table 1:** Baseline characteristics of atrial fibrillation patients compared among the 3 different antithrombotic regimens.

Variables	All (*N* = 3,148)	OAC alone (*n* = 2,258)	OAC+antiplatelet (*n* = 308)	Antiplatelet alone (*n* = 582)	*p* value
Age (years)	68.1 ± 10.8	68.4 ± 10.8	68.2 ± 9.6	67.0 ± 12.0	0.018^c^
Female gender	1,322 (42.3%)	1,017 (45.0%)	97 (31.5%)	218 (37.5%)	<0.001^a,b^
Time after AF diagnosis (yrs)	3.5 ± 4.4	3.6 ± 4.4	3.3 ± 4.3	3.2 ± 4.1	0.159
Atrial fibrillation					<0.001^a,b^
(i) Paroxysmal	998 (31.7%)	657 (29.1%)	121 (39.3%)	220 (37.8%)	
(ii) Persistent	603 (19.2%)	406 (18.0%)	75 (24.4%)	122 (21.0%)	
(iii) Permanent	1,547 (49.1%)	1,195 (52.9%)	112 (36.4%)	240 (41.2%)	
Symptomatic AF	2,421 (76.9%)	1,751 (77.5%)	222 (72.1%)	448 (77.0%)	0.102
History of heart failure	866 (27.5%)	578 (25.6%)	123 (39.9%)	165 (28.4%)	<0.001^a,c^
History of CAD	540 (17.2%)	227 (10.1%)	189 (61.4%)	124 (21.3%)	<0.001^a,b,c^
CIED	323 (10.3%)	224 (9.9%)	46 (14.9%)	53 (9.1%)	0.015^a,c^
History of ischemic stroke/TIA	578 (18.4%)	477 (21.1%)	61 (19.8%)	40 (6.9%)	<0.001^b,c^
Hypertension	2,232 (70.9%)	1,619 (71.7%)	242 (78.6%)	371 (63.7%)	<0.001^a,b,c^
Diabetes mellitus	819 (26.0%)	564 (25.0%)	126 (40.9%)	129 (22.2%)	<0.001^a,c^
Smoking	618 (19.6%)	383 (17.0%)	90 (29.2%)	145 (24.9%)	<0.001^a,b^
Dyslipidemia	1,822 (57.9%)	1,278 (56.6%)	228 (74.0%)	316 (54.3%)	<0.001^a,c^
Renal replacement therapy	38 (1.2%)	14 (0.6%)	8 (2.6%)	16 (2.7%)	<0.001^a,b^
Dementia	29 (0.9%)	19 (0.8%)	6 (1.9%)	4 (0.7%)	0.131
History of bleeding	308 (9.8%)	225 (10.0%)	47 (15.3%)	36 (6.2%)	<0.001^a,b,c^
History of PAD	41 (1.3%)	20 (0.9%)	12 (3.9%)	9 (1.5%)	<0.001^a^
CHA_2_DS_2_-VASc score					<0.001^a,b,c^
(i) 0	108 (3.4%)	60 (2.7%)	1 (0.3%)	47 (8.1%)	
(ii) 1	357 (11.3%)	215 (9.5%)	21 (6.8%)	121 (20.8%)	
(iii) ≥2	2,683 (85.2%)	1,983 (87.8%)	286 (92.9%)	414 (71.1%)	
HAS-BLED score					<0.001^a,b,c^
(i) 0	344 (10.9%)	334 (14.8%)	2 (0.6%)	8 (1.4%)	
(ii) 1-2	2,276 (72.3%)	1,657 (73.4%)	160 (51.9%)	459 (78.9%)	
(iii) ≥3	528 (16.8%)	267 (11.8%)	146 (47.4%)	115 (19.8%)	

Data presented as mean ± standard deviation or number and percentage. Abbreviations: OAC: oral anticoagulant; AF: atrial fibrillation; CAD: coronary artery disease; CIED: cardiac implantable electronic device; TIA: transient ischemic attack; PAD: peripheral arterial disease. ^a^Statistically significant (*p* < 0.05) OAC alone vs. OAC+antiplatelet. ^b^Statistically significant (*p* < 0.05) OAC alone vs. antiplatelet alone. ^c^Statistically significant (*p* < 0.05) OAC+antiplatelet vs. antiplatelet alone.

**Table 2:** Clinical outcome events per 100 person-years and absolute 2-year risk compared among antithrombotic regimens for each clinical outcome.

Regimens	Patients	Events	100 person-years	Rate per 100 person-years (95% CI)	Absolute 2-year risk (95% CI) (death as competing risk)
Ischemic stroke/TIA					
OAC alone	2,258	59	46.4	1.27 (0.97-1.64)	2.49 (1.90-3.21)
OAC+antiplatelet	308	11	7.2	1.53 (0.76-2.73)	3.30 (2.58-4.15)
Antiplatelet alone	582	29	12.9	2.25 (1.51-3.23)	4.35 (2.93-6.18)
Major bleeding					
OAC alone	2,258	110	46.4	2.37 (1.95-2.86)	5.05 (4.14-6.08)
OAC+antiplatelet	308	23	7.2	3.19 (2.03-4.79)	4.20 (3.38-5.15)
Antiplatelet alone	582	19	12.9	1.47 (0.89-2.30)	3.49 (2.37-4.94)
Death					
OAC alone	2,258	167	46.4	3.60 (3.07-4.19)	
OAC+antiplatelet	308	39	7.2	5.41 (3.85-7.40)	
Antiplatelet alone	582	53	12.9	4.11 (3.08-5.37)	

Abbreviations: CI: confidence interval; TIA: transient ischemic attack; OAC: oral anticoagulant.

## Data Availability

The dataset that was used to support the results and conclusion of this study are included within the manuscript. Additional data are available from the corresponding author upon reasonable request.
